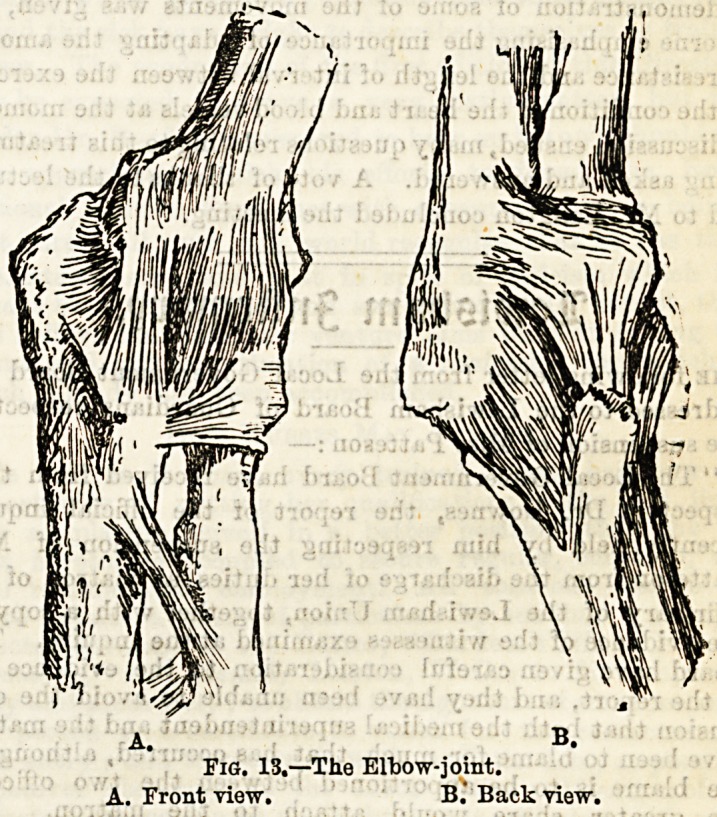# The Hospital Nursing Supplement

**Published:** 1895-02-23

**Authors:** 


### "The Hospital" Nursing Mirror.

Being the Extra Nursing Supplement of "The Hospital" Newspaper.

[Contributions for this Supplement should be addressed to the Editor, The Hospital, 428, Strand, London, W.C., and should have the word "Nursing" plainly written in left-hand top corner of the envelope.]

### CREATING A GRIEVANCE.

Attention has been called by some of our contemporaries to a supposed grievance in connection with the new Nurses' Home at the South-Eastern Fever Hospital. It is asserted, on the authority of certain junior nurses, that the Home was built and furnished for the use of the whole staff, and the grievance consists in their not all being admitted to it. We are officially informed that the Home was originally designed for the charge nurses only, but there proving to be sufficient bed-rooms for the first assistant nurses they were also accommodated there. The nursing staff numbers over a hundred members, whilst there are only eighty-two new bed-rooms, and therefore the second assistant nurses are placed in the apartments formerly used by the charge nurses; each having a separate bed-room and the use of dining and sitting-rooms, which served for the charge nurses until the new Home, described Jan. 27, in the "Nursing Mirror," was erected. Much has been done for the comfort of the staff at this hospital, amongst other things two wardmaids engaged to help the nurses in keeping up fires, carrying linen to the laundry, &c., during the night. If the discontented persons had addressed their complaints directly to the medical superintendent or to the committee, it would have been more courteous than to publicly attack an arrangement which they have apparently not troubled to understand.

### MISS GIBSON'S PAPER.

In the able paper on "Nursing in Workhouses and Workhouse Infirmaries" which Miss Gibson read at the Poor Law Conference held last week at the Guildhall, she remarked, "I do not think, in these days, there is much, or, indeed, anything that is new to be said about workhouse nursing. A great deal has been done, but very much remains to be done." Miss Gibson spoke of the broad lines existing at Liverpool, Manchester, and Birmingham, where "the nurses are given their proper position, are expected and encouraged to uphold the dignity of their office." In contrasting the large, well-managed workhouse infirmaries with the small country unions, the absence in the latter of night nurses was strongly condemned. "It seems absolutely necessary that a night nurse in an infirmary in which there is no medical officer should be a high-minded, fully-qualified woman." Miss Gibson made many practical suggestions regarding the training of probationers and the administration of the nursing department. Her personal experience at the magnificent workhouse infirmary at Birmingham gives special value to the suggestions of this Matron. Many will agree with her that "the remedy—the only drastic and complete remedy to my mind—must be in trained women inspectors, who are appointed to look into the nursing of the sick, especially into the arrangements for the women and children. . . ." Miss Gibson remarked that those who detract from the importance of infirmary nursing "are chiefly those who do not know its possibilities."

### WHO SHOULD INSPECT?

The demand for women inspectors of workhouses and infirmaries has been so often repeated that thoughtful people begin to consider how many trained nurses would be found ready to fill such posts if they were opened to them? Practical knowledge of hygiene and sanitation would certainly be required, and a thorough experience of the working of the Poor Law. The ideal inspector would probably be found in a fully-trained and successful workhouse infirmary matron, an intelligent lady who is personally acquainted with the difficulties of the workers in such institutions. Unless the salaries offered are sufficiently good to attract candidates of the highest qualification and character, even the appointment of women inspectors must fail to be "the complete remedy" which Miss Gibson claims for it.

### EAST GRINSTEAD COTTAGE HOSPITAL.

The Committee of the East Grinstead Cottage Hospital issued their sixth annual report at a meeting of subscribers which was well attended. The excellent work of Miss Oxtoby, the Matron, and her nurse was appreciatively mentioned.

### DISTRICT NURSES AT BIRMINGHAM.

The Birmingham District Nursing Society does not receive the substantial support which it appears to merit, its expenditure during the past year being in excess of its income. The Branch Home at Saltley is a valuable extension of the work of the society, and seems to be appreciated. St. Augustine's Clothing Club and the Solihull Centre of the Needlework Guild have supplied many useful garments, and free passes to the nurses have been generously granted by the Birmingham Central, the Birmingham and Midland, and the Birmingham and Aston tramway companies. Increased financial support will assuredly follow the announcement of the Society's needs made at the annual meeting.

### NURSING AT LICHFIELD.

The Mayor of Lichfield recently presided over a meeting held in the Guildhall to discuss plans for extending the work of the local nursing institution. It is reported that a sub-committee has been chosen to prepare a scheme for securing a nurses' home, with accommodation for accident and other emergencies; and also to consider the subject of obtaining financial support for the undertaking.

### THE TRAINED NURSE AT ELY.

The Bishop of Ely, as President of the District Nursing Association, took the chair at the annual meeting, which was held at the Palace. The report placed before the subscribers was a satisfactory one, and all the speakers warmly advocated the benefits conferred by skilled nursing of the sick poor in their own homes. Miss Whitehead, the present nurse, had attended a number of children suffering from bronchitis, and had paid altogether 2,535 visits to 127

patients since February, 1894. A number of chronic cases require constant attention at Ely, although the exceptional healthiness of the neighbourhood is said to limit the cases of acute forms of illness.

### SHEPTON MALLET.

Shepton Mallet District Hospital has been in existence twenty-five years, and its expenditure and income seem to be very evenly balanced. At the recent annual meeting the efficient and economical management of the matron, Miss Spurling, was heartily commended. A hundred and forty-eight patients were nursed during last year, seventeen of them being casualties.

### THE QUESTION OF RELIGION.

Discussions have been taking place lately with regard to the eligibility of Roman Catholics for admission to nurse training schools. It is doubtless true that many naturally prefer to enter institutions which are nursed by those of their own religion, but it seems generally conceded that in the case of candidates who give promise of making good nurses sectarian difficulties are rarely raised anywhere. It appears, from the report of the proceedings at the Select Vestry at Liverpool, that "sufficient education to write and to read doctors' directions" qualifies women for becoming probationers. Even in hospitals where a higher standard of education is maintained, Roman Catholics are generally welcomed equally with women of other religious denominations.

### KIND, BUT UNSKILLED.

The Kidderminster Guardians have decided not to continue the grants to the Nursing Institution of Wribbenhall, Bewdley, Stourport, and Kidderminster. Although five members voted in favour of securing the continuance of skilled nursing for the sick poor, twelve were against it. The kindness of the poor to each other was advanced as an argument against the need for trained nurses, the remarks of several persons present, notably of the ladies, showing that the principles of well-organised district nursing are little understood in the neighbourhood of Kidderminster.

### KILMARNOCK INFIRMARY.

A pleasant social evening was recently enjoyed by the nurses at the Kilmarnock Infirmary, various friends of the institution contributing to the musical programme. Several short speeches were made, all cordially testifying to the admirable work of the matron, Miss Bowman, and her staff.

### CHARITY IN DUBLIN.

In connection with the help given by the Dublin Coal Fund, Miss Dunn, General Superintendent of the Irish Branch of the Queen's Nurses, gives most cordial testimony to the assistance afforded by many local charitable societies. So much distress comes to the personal knowledge of the district nurses in Dublin that they have constant need to claim kindly co-operation in caring for the sick and poor.

### THE PRESIDENT AT BELLEVILLE.

President Faure devotes Thursday mornings to visiting charitable institutions in Paris. Last week Tenon Hospital at Belleville was chosen for inspection. After conversing with many of the patients the President was presented with a bouquet by one of them, and on departing he gave a donation of £24 to the manager "to improve the beef tea of the patients." It is to be hoped that a permanent improvement will take place, if, as seems implied by this gift, the diets are not of the best quality.

### A PLEASANT EVENING.

The St. Pancras Infirmary nursing staff spent a pleasant evening in their large new sitting-room last week. The first part of the entertainment was enjoyed by the night nurses, and when they went on duty the day nurses came in for their share of amusement. The neatness of the uniforms was as noteworthy as the charming decorations of the spacious room. Excellent music was performed by several of the nurses, and Miss Gethen recited a number of original stories of hospital life. An occasional social evening of this sort must make an agreeable variation in the daily routine of workhouse infirmary nursing.

### CAROLS AT SYDNEY.

A pleasant Christmas was spent by the patients in the fine wards of Prince Alfred's Hospital, Sydney. Fine palms, flowers, holly, Christmas bush, and evergreens were contributed by various friends, and the decorations, in which the patients took great interest, were peculiarly effective and graceful. One of the pleasantest features of the festival was the singing of carols on Christmas Eve, in which the matron, sisters, and some of the nurses were assisted by the organist, Mr. Percy Howe. They began in the verandahs, and traversed most of the wards and corridors of this handsome hospital. The patients were so much delighted with the charming performance that Miss McGabey (the Matron) and her nurses must have felt amply repaid for their exertions.

### SHORT ITEMS.

The recent examination of the London Obstetrical Society was successfully passed by Miss Baker and Miss Everton, of Guy's Trained Nurses' Institution; they had previously attended midwifery cases in connection with the Guy's Lying-in Charity, supervised by a midwife for a period of three months.—The thanks of the governors of the Farringdon Dispensary were given at the annual meeting for the daily services of a nurse from the Metropolitan and National Nursing Association.—A concert in aid of the Nursing Association was recently held in the Albert Hall, Gainsborough, under the auspices of the Saturday Hospital Committee.—Miss Helena Bockin has been made parish nurse at Sidcup.—The Drogheda District Nursing Association received £18 from the Walton-Leslie concert.—Lizzie B. Ebborall, described as a nurse at Nottingham County Asylum, was recently charged with obtaining goods from two drapers, not paying for them, and giving a false name. The magistrates gave the option of a fine in both cases, "to give her a chance of retrieving her character."—The Guardians have unanimously agreed to engage a night nurse for the sick ward at the Hereford Workhouse.—Miss Barker has resigned the post of lady superintendent of the Stockton-on-Tees and Thornaby District Nursing Association, on account of her approaching marriage; the committee appreciate her excellent work during the past two years.—A good record of work was shown at the annual meeting of the Bideford Nursing Society.

### Elementary Anatomy and Surgery for Nurses.

By W. McAdam Eccles, M.B., M.S., F.R.C.S., Lecturer to Nurses, West London Hospital, &c.

### VII.—THE PRINCIPAL JOINTS OF THE BODY; WITH THE CHIEF MUSCLES WHICH MOVE THEM.

A joint is the place where two or more bones are connected together, and is often termed an articulation. At the various joints the different bones are moved upon one another by

Fig. 11.—Vertical Section of the head of the Femur.

the action of muscles. There are three kinds of joints: (1) Movable, which are the most numerous, *e.g.*, the elbow; (2) yielding, where the range of movement is but slight, *e.g.*, between the vertebræ; (3) immovable, but few in number, *e.g.*, between the bones of the vault of the skull. The following different structures enter into the formation of most joints: (1) Bones; (2) cartilage; (3) ligaments; (4) synovial membrane. The portion of the bones where they come in contact is generally expanded, and if a section be made through this it will be seen to be composed of a honeycomb structure, termed cancellous tissue, which is covered by a thin layer of firm compact bone. (See Fig. 11.) The cartilage invests the end of the bone and gives it a very smooth surface. In some joints a special variety of cartilage, called fibro-cartilage, may occur as a kind of buffer between the bones, as is seen in the intervertebral joints. The ligaments are strong fibrous bands which serve to bind the bones together, and while being usually sufficiently loose to allow of movement, yet they limit in most cases excessive motion. The synovial membrane lines the interior of the joint, and secretes a fluid somewhat like unboiled white of egg, which lubricates the surfaces of the cartilages. This is termed synovia.

Various movements take place at joints, and these have been classified in four groups: (1) Angular movement, as

Fig. 12.—The Acromio-clavicular and Shoulder-joints.

seen in the elbow joint; (2) gliding, as seen in the carpal joints; (3) rotation, as between the radius and ulna; (4) circumduction; that is, a movement produced when all the angular movements possible are performed quickly one after another, as in the shoulder joint when the arm is swung round. The various voluntary movements of a joint are produced by the contraction of muscles which are attached to the bones. A short description of the more important movable joints of the body is necessary.

### The Joints of the Upper Extremity.

(1) The sterno-clavicular articulation. This lies between the inner end of the clavicle and the upper part of the sterum; it is divided into two halves by an inter-articular fibro-cartilage; the arm is attached to the trunk in part through this joint. (2) The acromio-clavicular joint is between the outer end of the clavicle and the acromion process of the scapula. (3) The shoulder joint (see Fig. 12) is the most freely movable joint of the body, and is termed a ball-and-socket articulation. The bones entering into it are the glenoid cavity of the scapula above, and the rounded head of the humerus below, each of these surfaces being covered with articular cartilage. The chief ligaments are the capsular, which surrounds the whole joint, but is very lax to allow free motion. Within this is the glenoid ligament around the margin of, and deepening the glenoid fossa. The long tendon

A. B.

Fig. 13.—The Elbow-joint.
A. Front view. B. Back view.

of the biceps muscle passes through the joints above the head of the humerus, and acts in the capacity of a ligament. (4) The elbow joint (see Fig. 13) includes the humerus above and the radius and ulna below. This is a hinge joint, the most perfect of its kind in the body. There is an interesting articulation between the radius and ulna, both at their upper and lower ends. The head of the radius rotates within the orbicular ligament on the ulna, while at the lower end the radius rotates around the head of the ulna. (5) The wrist joint has below the under surface of the lower end of the radius, and a fibro-cartilage beneath the head of the ulna, and above three bones of the first row of the carpus.

### Presentations.

An address and a pocket-case of instruments were presented to Staff Sister Marian La Fontaine on her retirement from the National Children's Hospital, Dublin, to enter on the study of medicine. The chair was occupied by Major-General Moncrieff, General Commanding Dublin District. Mr. L. Hepenstal Ormsby, F.R.C.S., the founder of the order, read the address, and said that Sister Marian La Fontaine was one of many able and efficient workers who had entered the Dublin Red Cross Order of Nursing Sisters, and earned respect and regard.

### Royal British Nurses' Association.

DR. BEZLEY THORNE'S LECTURE.

AN interesting lecture on the "Physical Treatment of Heart Disease" was given by Dr. Bezley Thorne on Friday evening at 17, Old Cavendish Street. Mr. John Langton occupied the chair, and, notwithstanding the severity of the weather, a large audience assembled. After giving an outline of the physiological effects of this method of treatment as elaborated by the Drs. Schott, of Bad Nauheim, and describing the constituent properties and therapeutic value of the baths and exercises, the lecturer cautioned nurses from rashly undertaking to conduct treatment apparently simple, but nevertheless fraught with danger, unless most scientifically administered. Not only must undue fatigue to the patient be avoided, but the following points must be carefully noted; the expression of the face, the movements of the nostrils, the appearance of sweat, the colour of the complexion, and the chest movements. The observation of such details demands great alertness and skill, therefore only highly trained women are suitable for the work. The administrator must also be expert in the use of the sphymograph and competent to report to the medical attendant the slightest deviation from the normal. Towards the conclusion of the lecture a demonstration of some of the movements was given, Dr. Thorne emphasising the importance of adapting the amount of resistance and the length of interval between the exercises to the condition of the heart and blood vessels at the moment. A discussion ensued, many questions relating to this treatment being asked and answered. A vote of thanks to the lecturer and to Mr. Langton concluded the meeting.

### Lewisham Infirmary.

THE following letter from the Local Government Board was addressed to the Lewisham Board of Guardians respecting the suspension of Miss Patteson:—

"The Local Government Board have received from their inspector, Dr. Downes, the report of the official inquiry recently held by him respecting the suspension of Miss Patteson from the discharge of her duties as matron of the infirmary of the Lewisham Union, together with a copy of the evidence of the witnesses examined at the inquiry. The Board have given careful consideration to the evidence and to the report, and they have been unable to avoid the conclusion that both the medical superintendent and the matron have been to blame for much that has occurred, although if the blame is to be apportioned between the two officers, the greater share would attach to the matron. The Board think that Miss Patteson was especially indiscreet in transferring nurses from an erysipelas ward to general wards on two occasions without having previously consulted the medical superintendent. Upon a review of the whole case, however, it appears to the Board that the questions which the inspector was called upon to deal with at the inquiry were of so trivial and petty a nature that it would be unreasonable to suppose that they could form causes of permanent disagreement between officers, the record of whose services hitherto has been of so satisfactory a character. They have therefore determined to defer their decision in the case for six months, removing in the meantime the matron's suspension and allowing her to resume her duties. The Board trust that this course may result in a good understanding being established between these officers, but at the same time they think it right to state that the good administration of the institution cannot be allowed to suffer from want of harmony amongst the officers, and that if further cause for complaint arises the officer who is shown to be in fault cannot be allowed any longer to retain office. The Board think it right to add that they have observed with regret evidence of want of courtesy on the part of the steward towards the matron, and they request that he may be cautioned as to his future conduct in this respect."

After a long discussion, it was resolved by the Guardians present at the last meeting

"That this Board of Guardians have received with regret the decision of the Local Government Board reinstating the the matron of the infirmary, and this Board petition the Local Government Board to reconsider that decision. The Guardians request the President of the Local Government Board to receive a deputation of the whole Board who will lay before him serious reasons for reconsideration of the whole matter. The Guardians feel so strongly on the subject that pending the reception of the deputation they have given orders that the suspended matron is not to resume her duties."

In accordance with this decision, a small deputation of Guardians was last week received by the Local Government Board, and the result of the interview will doubtless be made public at the next meeting of the Lewisham Board of Guardians. Until after this event any comments would be obviously out of place, but it is already rumoured that the recommendations of the Local Government Board published above are not likely to be materially altered.

### English Nurses at Hong Kong.

IN the wards where the Europeans are nursed at the Government Civil Hospital, Hong Kong, Christmas has been duly honoured. Although neither holly, box, nor laurel flourishes, and the weather in midwinter is warm, sunny, and bright, other shrubs furnished decorations sufficiently suggestive of Christmas at home. The "sailors'" and "policemen's" wards wore a most festal appearance, many gay flags being lent from ships lying in harbour, whilst quantities of fine chrysanthemums, obtained at trifling cost, added much to the general effect. On Christmas morning the nurses sang carols in the verandahs to the general delight and surprise of the patients, and afterwards service was held in the largest ward, in which all who were able took part. A real English Christmas dinner was followed by an excellent concert, and at half-past six a daintily-served tea concluded an entertainment which will long be remembered by the patients for whose pleasure it was arranged. A sad incident in the day was the admission of a poor Danish sailor with both femurs fractured (one compound) and a fractured skull, but of course, he was tended by doctor and nurses in a part of the hospital at some distance from that in which Christmas was in course of celebration. The Chinese hold the New Year as their chief annual festival—it is a movable feast, commencing in 1895 on January 26th. The firing of crackers is the chief pleasure indulged in, and the time in which this incessant noise pervades "China Town" is in Hong Kong limited by Government; in Canton and other towns the feast of the New Year lasts for a fortnight.

### Appointments.

[It is requested that successful candidates will send a copy of their applications and testimonials, with date of election, to THE EDITOR, The Lodge, Porchester Square, W.]

CHELSEA HOSPITAL FOR WOMEN.—Miss Mildred Heather-Bigg has been appointed matron of this hospital. She was trained at University College Hospital, and was afterwards ward sister at Charing Cross Hospital and matron at Bromley Cottage Hospital, Kent. Miss Heather-Bigg holds good testimonials, and we wish her every success in her new work.

JENNY LIND INFIRMARY, NORWICH.—Miss May Harding has been appointed Lady Superintendent of the Jenny Lind Infirmary. She received three years' training at King's College Hospital, London, and in November, 1892, was appointed Ward Sister at the Hospital for Sick Children, Pendlebury, Manchester. This post Miss Harding vacated to take up her new duties at Norwich, carrying with her many cordial good wishes.

ROYAL HANTS COUNTY HOSPITAL, WINCHESTER.—Miss Mary Mocatta has received the appointment of matron to this hospital. She was trained at University College Hospital, and was afterwards staff nurse there, and ward sister at Charing Cross Hospital. For nearly six years Miss Mocatta has been matron of the Royal Hospital for Children and Women, Waterloo Bridge Road, and she takes with her most hearty good wishes to her new work, in which we wish her every success.

### Burdett's Official Nursing Directory.

THE publication of a nursing directory of a strictly official and neutral character has long appeared to many of those who have considered the question to be an undertaking which would be directly advantageous to the nursing profession, and would at the same time afford to the public some measure of protection against those women who, with little or no training worthy of the name, advertise themselves as competent attendants upon the sick. It is a feature of this Directory that no charge will be made for the insertion of particulars.

### No Fees Should be Demanded.

We have all along felt it to be wrong to demand fees from nurses, even for registration, because a nurse has to pay pretty heavily in money, or in money's value, for her training. Her earnings are none too large, under the best of circumstances, and if registration, or the inclusion of her name in a directory be essential to her calling, she ought to gain these advantages without further payment, as one result of her training. Of late years there has been a growing tendency not only to levy fees upon nurses, but to increase the amount of those fees to an extent which is not only undesirable, but wholly unnecessary, if due regard be had to the public interests at stake and the claims which nurses have to recognition as zealous workers among the sick. Hence we welcome the present attempt to offer every nurse in the British Empire an opportunity of making her claims known to the members of the profession and the public who are likely to need her services, with the added guarantee that the qualifications which follow her name have been verified upon evidence which is beyond question. The kindly co-operation of matrons and nurses, which the compilers of "Burdett's Official Nursing Directory" invite, is, we are confident, already secured, because both parties must recognise the importance of co-operating to secure that the particulars given shall be both full and accurate. We are glad to notice that the compilers do not claim for the Directory any authority analagous to that possessed by the Medical Register, nor do they believe that the time has yet arrived when a rigid conformity in curriculum and period of training can be enforced upon those who desire to place their names on a directory of trained nurses.

### What this Directory Will Do.

What "Burdett's Official Nursing Directory" will do is to state the facts as nearly as they can be ascertained, and so to place every nurse in her right position according to her training and qualifications. It is further satisfactory to note that the right of excluding any names from this Directory, and declining to publish any particulars with reference to work, training, or other matters which appear to be insufficiently authenticated or otherwise unsuitable, is reserved. The object of the editorial committee is to secure justice for all, and the publication of "Burdett's Official Nursing Directory" will enable every nurse in the profession to be independent, and free from pressure of all kinds, because she will now be enabled to make her claims understood and recognised by the members of the profession and the public upon whom she has to rely for continuous work. We are informed by the compilers that many thousands of nurses have been personally requested to supply up to date particulars of their training and qualifications, a request which we have no doubt will be promptly responded to, owing to the strictly official and neutral character of the present attempt to render a lasting service to every nurse throughout the British Empire.

### Important to Remember.

We are asked to direct the attention of nurses to the fact that on the form provided for the insertion of particulars of their qualifications and training, blank spaces are left for the signature of some responsible person to vouch for the correctness of each entry. No form should be returned until it has been properly tested by the addition of the signature in question, or failing such signature, a nurse may transmit the orginal certificate of training. Matrons and lady superintendents are invited to make suggestions, and to co-operate in securing that the information concerning the training schools over which they preside shall be full, impartial, and accurate. There can be little doubt that in the interests of different institutions the governors will desire that the statements made in "Burdett's Official Nursing Directory," both with reference to its nursing school and to individual nurses who have been trained within its walls, should be as complete and authentic as possible.

### Nurses in their Own Defence.

We commend this Directory to the attention and support of nurses in defence of their rights and independence. We anticipate that the committee of medical men and matrons who are associated with Mr. Burdett may rely on the co-operation of the large army of trained nurses who wish to see their profession placed upon a sure foundation, to make this work a trustworthy guide to the numerous body of women desirous of entering the nursing profession, and to the public at large, which seeks its guarantee that those employed to attend the sick have a right to describe themselves as trained nurses. The production of the first edition of this Directory may be expected to prove a very costly matter, but this burden Mr. Burdett is prepared to bear on his own shoulders. The appreciation which his efforts to found the Royal National Pension Fund met with encourages him to believe that nurses all over the world recognise that he has their interests at heart, and that in spite of criticism which has always been ill-natured and sometimes hardly honest, they will believe that this new venture aims only at raising the status and public estimation of a profession for which so much already has been accomplished.

### How Nurses May Benefit.

Be this as it may, every trained nurse has now the opportunity of putting her qualifications in the best light, and so placing herself in a better position than she has ever heretofore occupied to secure regular, constant, and well remunerated employment. All that she has to do is to obtain a form of particulars by sending a stamped directed envelope to the Editor, "Burdett's Official Nursing Directory," 428, Strand, London, W.C.; to fill it up carefully and accurately; to obtain the signature of some responsible person or a certificate to vouch for the correctness of each entry; and to return it with as little delay as possible. Whatever else may be the effect of the publication of this Directory, there can be no doubt that it will place each nurse in a better position to earn her living and maintain her independence than she has ever before occupied.

### Legal Intelligence.

### PRIVETT V. CHASTON.

THIS was an action brought at the Bloomsbury County Court on January 30th, 1895, by Miss Elizabeth Frances Marion Hill Privett against Miss Jessica Iris Chaston to recover the sum of £4 1s. for salary earned and expenses incurred whilst employed as a nurse at the defendant's Home for nurses at 36, Devonshire Street, Portland Place. Mr. William Young appeared for the plaintiff; the defendant did not appear. The plaintiff's case was that she was engaged by the defendant on March 1st, 1894, as a nurse in her Home at a salary of £10 per annum for the first six months, and afterwards at the rate of £14 per annum, with board and lodging, and expenses of travelling to patients found. The plaintiff continued working for the defendant until October 20th, 1894, up to which time she had only received £3 out of £7 1s. due to her. She had made repeated applications for the balance but had not been paid. Judgment for £4 1s. and costs was given for the plaintiff.

### The Meath Hospital and County Dublin Infirmary.

A Visit to the Wards.

The Meath Hospital, which stands in its own grounds and is situated about five minutes' walk from St. Patrick's Cathedral, is irregularly built and of somewhat gloomy aspect externally, but this impression disappears as the visitor steps into the spacious tiled hall. It is adorned by busts of distinguished physicians, at different times connected with the hospital, and is particularly bright and attractive.

One of the staff sisters having kindly undertaken, in the absence of the lady superintendent, to act as guide, we visited the pharmacy—a model of neatness and convenience—and then we descended to the spacious kitchen. The roof of this apartment is supported by groined arches, which gives a somewhat mediæval effect, although in other respects the culinary department is essentially modern. Visiting the operation theatre on our ascent we passed in turn through the accident, surgical, and medical wards, which occupy respectively the first, second, and third floors. Each is provided with a bright little kitchen, newly tiled within the last year, and the children's ward, added to the hospital in 1864, is cheerful and pretty, but only two or three small patients were in possession of it, the numbers admitted being reduced as far as possible during the summer months. The little cots had been lately repainted by the hands of the sister in charge. She had chosen scarlet enamel for the purpose, evidently with a view to pleasing the tastes of the little people, and the effect was excellent.

Passing along the corridor we next entered the surgical ward of the "John]Barber Wing," a magnificent addition to the accommodation of the hospital, completed in 1888, and opened by his Excellency the Marquis of Londonderry, at that time Lord Lieutenant of Ireland. This is the finest portion of the hospital, the wards which it contains being larger, loftier, and better ventilated than those in the main building, dating from the middle of last century. Alike in old as in new, however, cleanliness, order, and comfort appear to prevail.

Training of the Nurses.

The nursing staff of the hospital is entirely drawn from the Red Cross Nurses' Home in Harcourt Street, where, in return for payment of a fee of £50, one year's training is given. At the end of this period an examination is held, probationers who pass satisfactorily being drafted as sisters into the Meath or Children's Hospital, Harcourt Street, if vacancies permit and the lady superintendent approve. Those who are twice unsuccessful in passing the prescribed examination are obliged to leave.

The staff sisters only are resident in the Meath Hospital, the probationers returning to the Harcourt Street Home at nine p.m., unless on night duty; their meals, however, are taken in hospital. Each of the sisters enjoys the comfort of a separate bed-room, and the one we saw was a dainty little apartment. A tiny little slip of a room is known as "the probationers' tea-room"; the general dining-room we inspected downstairs. A sitting-room in which the sisters might meet sociably when off duty appears to be wanting, but perhaps the pretty tennis ground devoted to their use affords sufficient compensation in the summer.

We must not omit to mention a small ward set apart for private patients, the charge being two guineas per week inclusive for the entire room, or one guinea if the patient is willing to share it with another. The ordinary dietary of of the patients includes eggs for breakfast, and alternate roast mutton and boiled beef, with vegetables, for dinner, varied by fish on Fridays. Of course, under medical directions, beef-tea, chicken, &c., can be substituted.

The out-patients' dispensary and consulting-rooms are entirely detached from the main building, as is also the infectious diseases wing, which is at present being rebuilt and extended, while the sanitary arrangements of the entire hospital have been perfected during the past twelve months. The total number of in-patients treated during the past year was 1 367; out-patients, 15,220. Everything seems to promise a long continuance of the progressive usefulness which the hospital has now so worthily accomplished for one hundred and forty years.

### The Book World for Women and Nurses.

[We invite Correspondence, Criticism, Enquiries, and Notes on Books likely to interest Women and Nurses. Address, Editor, The Hospital (Nurses' Book World), 428, Strand, W.C.]

Surgical Nursing. By Bertha M. Voswinkel. (Published by Messrs. P. Blakiston, Son, and Co., 1012, Walnut Street, Philadelphia. Price $1.00.)

This little volume contains some 163 pages of letterpress, an index, and a number of illustrations, the latter representing instruments, splints, bandages, and other surgical appliances. The book purports to have been written "for that large body of nurses whose technical education is not sufficiently advanced to enable them to thoroughly master the subject of general nursing in all its branches. . . ." Unluckily the author has not been particularly successful in adapting her teaching to those for whom it is intended. The subjects are not dealt with systematically, and remind us forcibly of the notes from a probationer's pocket-book, jotted down for her own private use. "Surgical Nursing," being designed for a larger public, contains elements of danger if looked upon as a guide for partially trained nurses. For instance, in the directions for the prevention of bed sores we read, "If, in spite of all precautions, a bed sore forms, use broad strips of adhesive plaster, carried across the back, to relieve pressure, covering in the ulcer and the surrounding skin several inches above and below." As no other advice is given, the reader may be excused for imagining that the whole treatment of a bed sore consists in not only coating a large surface of skin, but also the wound with plaster alone, neither dressing for the wound nor advice from the doctor being mentioned. Even the title, "Surgical Nursing," becomes inappropriate on pages in which such diseases as pneumonia, uremia, croup, and Bright's are included. The doctor is so seldom spoken of that the reader is constrained to believe that such directions as the following are addressed to the nurse. After learning that "hæmorrhage from the stomach is rare, but sometimes occurs, taking place in gastric ulcer or cancer of that organ." . . . "if bleeding be from the stomach give tannic acid, and if from some organ remote from the stomach give gallic acid. The dose is gr. x., repeat in twenty or thirty minutes." "Cold keys or pennies" dropped down the back are named with some approval as "old and simple treatment" for epistaxis. But more heroic measures are prescribed in hemoptysis, and we commiserate the unhappy sufferer to whom the author of "Surgical Nursing" recommends "a teaspoonful of salt upon the tongue, and direct the patient to swallow it. It is sometimes well to add a little vinegar. Ergot may be given in doses of from 30 to 60 drops." The directions for the treatment of fractures and dislocations are suggestive of infinite risks to patients if followed by the inexperienced women for whom this book is ostensibly compiled. The most commendable sentence in the chapter on dislocations and fractures comes at the end, and advises that in these cases "the nurse must be extremely careful in assuming responsibility; it is far better to do too little than too much." Massage and the vindication of its use is condensed by Miss Voswinkel into some four and a half small pages, and although the operator is warned to be gentle and to use the utmost care in giving massage after a fracture, she is also told "in all sprains use friction, kneading, rolling, and light percussion." The book is well got up and printed, and the illustrations make us regret that the writer did not content herself with giving a simple explanation of each one of them, rather than attempting to deal, even superficially, with a number of extraneous subjects in so limited a space.

### Everybody's Opinion.

[Correspondence on all subjects is invited, but we cannot in any way be responsible for the opinions expressed by our correspondents. No communications can be entertained if the name and address of the correspondent is not given, or unless one side of the paper only be written on.]

### THE UNAUTHORISED ADVERTISEMENT NUISANCE.

THE DEPUTY MATRON of a large Hospital in the Midlands writes to say: My experience *re Nursing Record* advertisements has been similar to that of the matron of a large institution in the North, whose letter appeared in a recent issue of your paper. Some months ago I advertised once in THE HOSPITAL for a re-engagement. I received several answers, and also obtained the appointment I now hold through the medium of your paper. Six weeks after the date of its appearance, when I had been established in my present work about a month, on taking up the current number of the *Record* I found my advertisement copied therein; and on reference to back numbers discovered that it had been running for five or six weeks. I at once wrote requesting withdrawal. The insertion had been fruitless. I unite with your correspondent in strongly condemning this practice (?) of the *Nursing Record*. The insertion was made without my authority, or the authority of anyone else connected with me, and no payment was made for it.

### PRIVATE NURSES AND PRIVATE INSTITUTIONS.

"NURSE B." writes: I read M. L. Canning's letter to you with surprise and indignation. I think an old saying we have in my county, "Well, if the cap fits wear it," might be rightly remembered in this case. This outburst of temper against an editor, whom nurses know to be their true friend, is both absurd and unjust. I, too, am a subscriber and a reader of THE HOSPITAL since its first number, and I am also a nurse of 20 years' standing. I have worked for many of the most eminent men in the profession, amongst all classes, and am employed now by one of the greatest specialists of the present day. I have every reason to believe that he is pleased to consider me one of his best nurses, whilst testimonials received in the course of last year show abundant appreciation of my services. I am a long way beyond 30, and in my opinion nurses are better over 30 for the private work. So much responsibility frequently lies entirely upon the nurse. It is very different to being in a hospital where there is a sister and the medical staff to appeal to. So, after many years of varied experience, and what most would call a successful career, I am convinced that no one should become a nurse with a view to making a fortune. It is only a love of the work that can make a really good nurse. Without it, discredit is often brought on a noble calling.

### THE MASSAGE QUESTION.

MR. E. LUKE FREER, F.R.C.S., writes: It is doubtful whether the articles which appeared in the *British Medical Journal* on this subject have not so far been productive of more harm than good, for I understand that many of the evils exposed in these articles still continue to exist in London, whilst a large number of honourable workers both here and in the provinces have found their occupation almost gone. Such a result is financially disastrous to themselves and at the same time prejudicial to the public. Simultaneously with the publication of these articles the opportunity presented itself for eliminating existing charlatanry, and at the same time placing upon a firm and scientific basis the practice of massage. This, however, has not been done, doctors and reputable operators appearing too panic-stricken to come forward in its defence. Months before these sensational articles appeared I drew attention to the abuses which were very generally known, and suggested an organisation somewhat on the following lines: An association of operators on a similar basis to the Royal British Nurses' Association, with—(1) A president, a layman or laywoman of well-known position in society. (2) Vice-presidents and council consisting of leading members of the profession and others whose *bona fides* were undoubted. (3) A number of examiners in different centres to investgate the fitness of workers before enrolment, irrespective of their sources of instruction, the fees for such examaninations to be paid by the Association. (4) The signature of two medical men of repute to each application for membership. (5) A strict code of rules for the government and conduct of members, to which each candidate for membership must subscribe. (6) Subscriptions from members annually to cover expenses. This is roughly the scheme upon which I would suggest that the conduct of massage work should be placed, and with that end in view I submit it to readers of THE HOSPITAL. Having to make use of massage daily in my practice I care not whether operators have certificates or not, so long as they are efficient; but in the interests of reputable workers as a body, I feel that something ought to be done to remove from them the stigma which appears at present attached to good and bad alike. I have been supplied with a copy of rules of the "British Massage Association" which are very exhaustive but not on the lines I have sketched, for I fail to see the names of any leading medical man or laymen in connection with the organisation, and without these such an association cannot secure confidence.

[Our correspondent seems unaware that there already exists the Society of Trained Masseuses, of which particulars can be obtained from Miss Angela Arthur, hon. secretary, address, 12, Buckingham Street, Strand, London.]

### HOW TO BECOME A NURSE.

MISS HONNOR MORTEN, Ivy Hall, Richmond, Surrey, writes: "I should be much obliged if you would allow me through your columns to state that a 3d. edition of 'How to Become a Nurse' is in the press, and that any corrections or additions received by February 25th will be attended to. Circulars have been sent out widely announcing the fact, but to some no answers have been received, and possibly some institutions may have been missed by mistake."

### Notes and Queries.

### Queries.

(80) *L.O.S.*—How can a certificated nurse best obtain the L.O.S. diploma with least expense?—*Nurse A.*

(81) *Training.*—I am anxious to obtain some training as a nurse, and shall be glad of advice.—*P.*

(82) *Six to Eighteen Months.*—Where can a young lady get six to eighteen months' hospital training to prepare her for a situation as under matron in a home or orphanage?—*T. A. C.*

(83) *Cottage Hospitals.*—Can you give me particulars as to organisation of cottage hospitals?—*Ireland.*

(84) *Nursing.*—Can you recommend me a book on general nursing?—*Holly Bush.*

(85) *Probationer.*—Am I too short to be trained. I am 4 feet 4 inches?—*Nanine.*

### Answers.

(80) *L.O.S. (Nurse A.)*—Consult the hon. secretary of the Midwives' Institute, 12, Buckingham Street, Strand, London, sending stamped addressed envelope for reply.

(81) *Training (P.)*—We cannot advise you unless we know how long a time you wish to devote to "some training," also your age, and other particulars.

(82) *Six to Eighteen Months (T. A. C.)*—It would be wise to go in for a complete hospital training and secure a certificate, and the only other plan is to be a paying probationer for the shorter period; but then you would not be a trained nurse. Read "How to Become a Nurse," by Honnor Morten. Published 428, Strand.

(83) *Cottage Hospitals (Ireland).*—Order "Cottage Hospitals," by Henry C. Burdett, published by Scientific Press, 428, Strand.

(84) *Nursing (Holly Bush).*—See answer to query (76) in last week's HOSPITAL. Either Lewis's "Theory and Practice of Nursing," 3s. 6d., or Miss Isabel Hampton's "Nursing," which is a larger work, might suit you.

(85) *Probationer (Nanine).*—You had better call on the secretary of the Workhouse Infirmary Nursing Association, 6, Adam Street, Strand, and ask her.

### Minor Appointment.

KNOTTINGLEY, YORKSHIRE.—Miss Margaret M. E. Hey, who was trained at the Evelina Hospital for Sick Children and University College Hospital, has been appointed District Nurse at Knottingley.

## Figures and Tables

**Fig. 11. f1:**
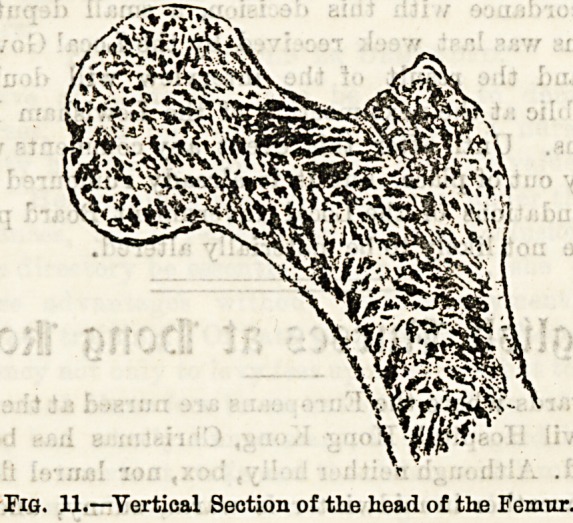


**Fig. 12. f2:**
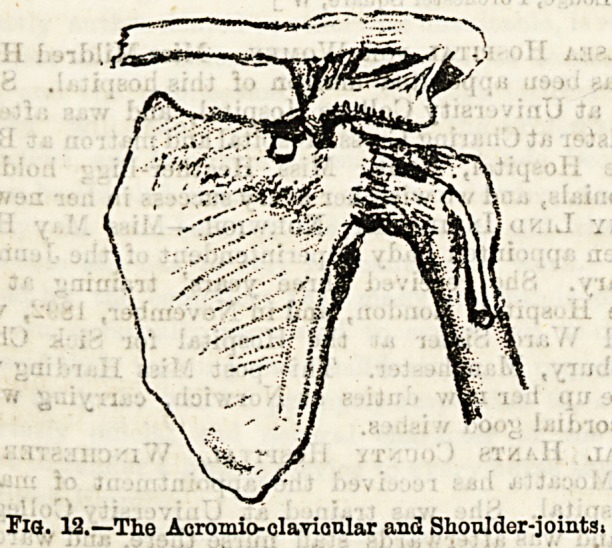


**Fig. 13. f3:**